# Diagnostic Efficiency of Multidetector Computed Tomography in the Evaluation of Clinically Equivocal Cases of Acute Appendicitis with Surgical Correlation

**DOI:** 10.7759/cureus.2249

**Published:** 2018-03-01

**Authors:** Jawaid Iqbal, Raza Sayani, Misbah Tahir, Syed M Mustahsan

**Affiliations:** 1 Radiology, Liaquat National Hospital and Medical College; 2 Department of Radiology, The Aga Khan University Hospital, Karachi.; 3 Emergency Medicine, The Aga Khan University Hospital, Karachi.

**Keywords:** acute appendicitis, diagnostic efficiency, computed tomography

## Abstract

Acute appendicitis is one of the most frequent causes of lower abdominal pain and requires immediate surgical intervention. The diagnosis often poses a lot of challenge even to experienced surgeon. Those patients with equivocal symptoms may require different imaging modalities like radiography, contrast examination and ultrasound with limited utility. Multidetector computed tomography (MDCT) used in suspected acute appendicitis has, however, resulted in improved diagnostic accuracy and also reduction of negative surgeries.

Objective

We intend to determine the diagnostic efficiency of MDCT in clinically equivocal cases of acute appendicitis correlating it with surgical/histopathological findings.

Materials and methods

A group of 116 patients was included in this study. Spiral MDCT was performed in all these cases after administration of oral and intravenous contrast. All these patients underwent surgery and the CT findings were correlated with histopathology. Out of these 116 patients, 60 patients were male and 56 female. The age range was from three to seventy years and mean age was 28+1 years.

Results

The results proved that MDCT had a sensitivity of 97.5%, specificity of 97.0%, and accuracy of 97.4% for the diagnosis of appendicitis with one false positive and two false negative cases. The study showed 100% accuracy in diagnosing acute appendicitis in children. In 33 patients, an alternate cause was identified with CT. The alternate diagnosis made on CT findings was consistent with the final diagnosis in 27 (81.8%) of 33 patients in whom there was no evidence of acute appendicitis. The clinical diagnosis disagreed with the CT diagnosis in six patients (18.18%).

Conclusion

Our study verifies that MDCT plays an important role in evaluation and consequent management of equivocal cases of acute appendicitis. MDCT is also able to diagnose appendicitis or detect alternative diagnosis in pediatric population.

## Introduction

Acute appendicitis is one of the most frequent causes of lower abdominal and right lower quadrant pain. This requires immediate surgical intervention in the form of appendectomy, which is one of the most common surgical procedures. The diagnosis often poses a lot of challenge even to experienced surgeon as several other gynecological, gastrointestinal and genitourinary conditions can have similar presentation [1,2]. Those patients with equivocal symptoms and without acute appendicitis could not only be spared of unnecessary cost but also of the associated morbidity of surgery if proper imaging and appropriate evaluation were done beforehand. In contrast, the morbidity associated with missing or delaying the diagnosis has serious consequences including appendicular perforation, peritonitis, abscess formation, sepsis, and can even cause death [3,4].

The clinical diagnosis of acute appendicitis has around 20% false-positive and a similar false-negative error rate [5, 6]. These patients will have equivocal clinical findings and/or laboratory test results thus making the diagnosis difficult [7-9]. To overcome this error rate different imaging modalities like radiography, contrast examination and ultrasound have been used with limited utility. Multidetector computed tomography (MDCT) used in suspected acute appendicitis has, however, resulted in improved diagnostic accuracy and also reduction of negative surgeries [10,11].

The purpose of this study was to determine the diagnostic efficiency of non-focused contrast-enhanced spiral CT abdomen to confirm or exclude appendicitis in patients who presented with equivocal signs and symptoms of appendicitis.

## Materials and methods

This was a comparative cross-sectional study performed at Departments of Radiology, Surgery and Pathology for a period of eight months. The sampling technique used was purposive with patients presenting either to the emergency department or outpatients’ clinics with periumbilical and/or right lower quadrant pain.

Patients of all ages presenting with periumbilical and/or right lower quadrant pain with pyrexia and leucocytosis with initial ultrasound examination yielding results inconsistent with the clinical presentation were included.

Patients with prior appendectomy, acute appendicitis confirmed on ultrasound, pregnant females, those who were unable to receive either oral or intravenous contrast material, those who were discharged without surgery and patients with clinically unequivocal cases of appendicitis undergoing laparotomy without imaging were excluded.

Initially, 123 patients were included in the study. Out of these, seven patients were excluded from the study. Two patients had the history of appendectomy, IV contrast could not be administered to two patients having the history of chronic renal failure and were on dialysis and three patients were discharged without surgery. So, finally 116 patients were included in the study. Out of these, 60 patients were male and 56 female. The age ranged from three to seventy years and mean age was 28+1 years.

All patients were restrained from taking anything orally. All CT scans were performed on Toshiba Asteon, Multislice CT Scanner. Opacification of the gastrointestinal tract was achieved through the oral and rectal administration of 3% diatrizoate meglumine solution (Gastrografin). Scans were performed before and after administration of I/V contrast. The abdomen was helically scanned during a single breath-hold (pitch of 1.5:1, 120 kVp, 240–320 mAs) from the dome of the diaphragm up to the symphysis pubis at an 8-mm collimation. A total of 1.5–2.0 ml of iodinated contrast was injected per kilogram of body weight, with doses based on age and weight with Medrad Vistron CT power injector system @2.0–3.0 ml/second. Informed consent was obtained from all patients. The image interpretation was done on the basis of primary and secondary diagnostic criteria for acute appendicitis. Acute appendicitis was labeled on CT scans if three or more of the following criteria were present.

**Figure 1:**
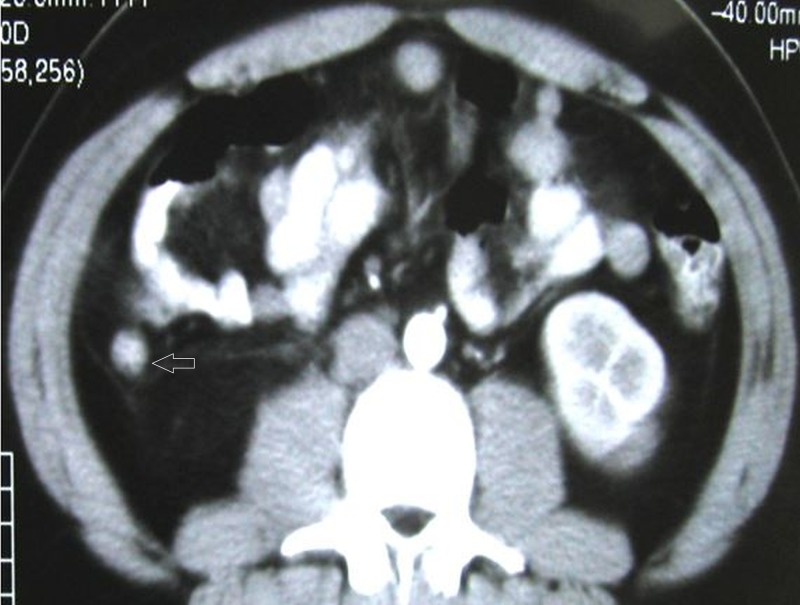
Acute appendicitis in a 21-year-old male. Axial contrast enhanced image showing dilated appendix measuring 11 mm (Arrow) in maximum thickness with enhancement of its walls.

Appreciation of a thickened/distended appendix which is >6 mm whether it is with or without mural thickening and enhancement (Figure 1).

**Figure 2:**
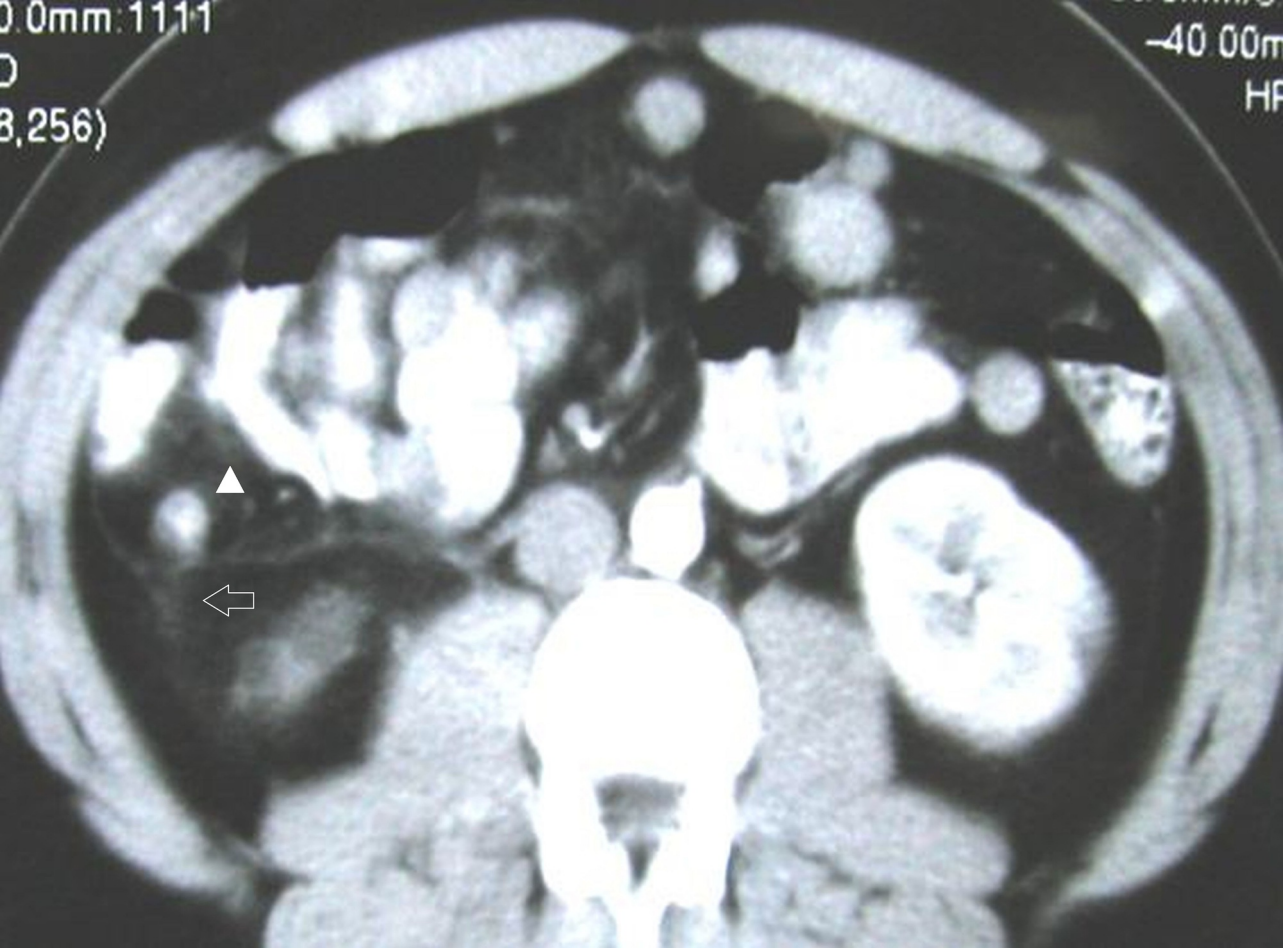
Acute appendicitis in a 41-year-old male. Axial contrast enhanced image showing periappendiceal fat stranding along with thickening of right Gerota's fascia (Arrow) and a dilated appendix (Arrowhead).

Periappendiceal fat stranding which is represented by progressively increased haziness and linear areas of high attenuation in the periappendiceal fat (Figure 2).

Secondary diagnostic criteria included the presence of appendicolith, periappendiceal abscess or phlegmon, mural thickening of the cecum, enlarged lymph nodes and extra-luminal air.

The scans were also evaluated for any other alternative diagnosis for the cause of symptoms. No case was lost to follow-up. In all selected 116 cases, the findings seen on the CT scans and surgical/pathological findings were collected and proforma was filled for each patient and they were compared with surgical and histopathology findings. Histopathological findings were taken as a gold standard for the diagnosis of appendicitis. Alternative diagnoses were evaluated in CT as well.

Data initially collected on proforma was then analyzed on SPSS Version 20. Different percentages and frequencies were obtained. Sensitivity, specificity, positive predictive value, negative predictive value, and accuracy were calculated on SPSS.

## Results

The results of our study showed that all patients had histopathological diagnosis of either acute appendicitis with or without associated findings or any of the alternative diagnoses after the CT scan. Correct assessment for acute appendicitis was made in 113 of 116 scans (97%). Incorrect assessment was made in three scans (3%). Of the 113 scans correctly evaluated, 80 were true positive for acute appendicitis and 33 were true negative, i.e., alternative diagnosis.

Of the three CT scans proven to be incorrectly evaluated, acute appendicitis was falsely interpreted as positive in one patient and as negative in two patients. One case that turned out to be false positive was of a 60-year-old female who had mucinous cystadenocarcinoma of appendix, a much rarer diagnosis. The cystic component of the mass was misinterpreted as the fluid-filled bowel not opacified with contrast.

In two false negative cases, the appendix was within the upper limits of the normal range of thickness. Marginal periappendiceal stranding was confirmed on retrospective analysis of the scans after the surgico-pathological outcome was known.

The diagnosis of acute appendicitis was made on the basis of presence or absence of major and minor criteria as discussed previously. In 80 patients with positive CT findings for acute appendicitis, the dilated non-opacified appendix (>6 mm in diameter) was visualized in 74 patients (92%) as shown in Figure 1 and not visualized in six patients (7%). In these six patients, an abscess in the RIF suggested a ruptured appendix.

In 33 patients with negative CT findings for acute appendicitis, normal appendix was seen in five patients (15%) and not seen in 28 patients (85%). In three out of the five cases in which normal appendix was seen, it was filled with contrast material, air was seen in its lumen in one case and in one patient no air or contrast was seen within the lumen but the appendix was of normal size.

Right lower quadrant fat stranding was a strong predictor of appendicitis. Among 80 patients with positive CT findings for acute appendicitis, moderate or severe stranding was identified in 53 patients (66%) as shown in Figure 1. An appendicolith was detected at CT in 11 (14%) patients, with an enlarged appendix in seven patients, and classical acute appendicitis, periappendiceal abscess/phlegmon were identified on CT scan in 14 cases (17%). Extra-luminal air was seen in two patients (3%) in case of perforation of the appendix along with a complex mass representing periappendiceal abscess or phlegmon. Abnormal cecal wall thickening was labeled on comparison with the normal wall thickness of ascending colon distal to the cecum and was seen in 23 patients (29%). Isolated, enlarged lymph nodes (>1.5 cm) in the pelvis were seen in 16 patients (20%) along with other findings for acute appendicitis especially in pediatric age group.

In 80 of 82 patients, acute appendicitis was diagnosed correctly on MDCT examination (sensitivity, 97.56%). It was correctly excluded prospectively in 33 of 34 patients (specificity, 97%). Overall accuracy for diagnosing acute appendicitis was 97.41%. The positive and negative predictive values were 98.76% and 94.28% respectively seen.

In 33 (28.4%) of 116 patients, alternative diagnosis was made with MDCT, including gynecologic abnormalities making up the largest proportion in 19 (57.5%) patients. Out of the nine adnexal masses reported, the possibility of ectopic gestation was successfully raised in three cases. Colonic pathologies were seen in four (12.1%), small bowel disease in six (18.1%), renal infection with calculi in three (9%) and acute cholecystitis was reported in one case (3%). Of the true-negative cases, the alternative diagnosis based on CT findings matched with the final diagnosis in 27 (81.8%) of 33 patients without acute appendicitis. The clinical diagnosis disagreed with CT diagnosis in six patients (18.18%).

## Discussion

Acute appendicitis may be a relatively straightforward diagnosis if it presents with typical signs and symptoms or when a distended, non-compressible thick-walled appendix is identified on ultrasound examination but the diagnosis may be more challenging when signs and symptoms are equivocal as in mild forms of appendicitis, where there may be minimal dilatation, less periappendiceal inflammation, small amount of retroperitoneal fat and in patients with significant pericaecal inflammation due to ruptured appendix [12,13].

MDCT has been shown to be highly precise in the diagnosis of acute appendicitis, though there is no consensus about which technique is ideal [14-16]. In our study, we scanned the entire abdomen and pelvis by using non-focused contrast-enhanced technique. This technique was also used by Balthazar et al. [17] and uses thin-section (5 mm) imaging through the pelvic region and thicker images in the upper portion of the abdomen. The principal reason for this approach is to pick other conditions which mimic appendicitis and have similar presentation [13,18].

IV contrast usage is important to patients with suspected acute abdomen as it improves the ability to identify the inflamed appendix, and to establish alternative diagnoses [18]. IV contrast also allows better evaluation of wall enhancement of appendix, differentiation of pelvic blood vessels from a retro-cecal appendix [19]. Contrast material cost and reaction risks are definite disadvantages [20,21], though no significant contrast reaction was seen in our study.

Studies with CT protocols without oral or per-rectal contrast agents have emphasized the time saved and patient discomfort [13,15]. Our patients did not complain of contrast intolerance or discomfort.

Rao et al. [19,22] used limited CT of the lower abdomen after oral and per-rectal contrast material administration, the technique used by us differed in several aspects as they included all patients clinically suspected of having acute appendicitis, whereas we examined only those patients who presented with equivocal sign and symptoms of acute appendicitis. CT was not performed in clinically obvious cases of acute appendicitis as the referring surgeons did not expect an added advantage and feared increased complications.

Thin-section focused helical CT was used by Lane et al. for the detection of suspected acute appendicitis and reported detection rate of 100% [23]. We were able to identify abnormal appendix in 74 (92.5%) of the 80 patients with acute appendicitis. We doubt the use of limited examination as a wide range of genitourinary and gastrointestinal conditions which mimic acute appendicitis and may be missed [24].

The major imaging findings of acute appendicitis at MDCT were based on thickened appendix measuring greater than 6 mm and periappendiceal inflammation [13,15]. Ours as well as the study by Rao et al. shows that periappendiceal inflammation is the most sensitive sign of acute appendicitis. An enlarged appendix of greater than 6 mm was 93% sensitive and 100% specific for acute appendicitis [25,26]. Additional findings on CT scans in acute appendicitis include cecal wall thickening, appendicoliths, and periappendiceal fluid collections [25].

One false positive case is our series was of a 60-year-old female who was diagnosed mucinous cystadenocarcinoma of appendix on histopathological examination, a much rarer diagnosis. The cystic component of the mass was misinterpreted as the fluid-filled bowel not opacified with contrast. 90% of all appendiceal tumors are carcinoids but lymphomas and carcinomas can also involve the appendix [26,27]. Most are found incidentally at surgery for another procedure or for appendicitis. Metastases are exceedingly rare [28]. In two false negative cases, the diameter of appendix was at the higher limit of normal range of thickness and there was marginal periappendiceal fat stranding on retrospective review.

Our study showed 100% accuracy in diagnosing acute appendicitis in children. Studies have also shown decrease in the rate of perforation in children who underwent CT as compared to those who did not (15% vs 23%). Also, a significant decrease in the negative appendectomy rate was noted in children with suspected acute appendicitis who underwent CT as compared to those who did not (6% vs 12%) [24,29].

In our study in 33 (28.4%) of 116 patients, an alternative diagnosis was made with CT, including gynecologic abnormalities in 19 (57.5%), colonic pathologies in four (12.1%), small bowel disease in six (18.1%), renal infection with calculi in three (9%), and acute cholecystitis was reported in one case (3%). This was in agreement with the study by Federle et al. and Rao et al. [23].

## Conclusions

The study shows that non-focused contrast-enhanced multi-detector CT is highly accurate in diagnosing acute appendicitis or suggesting an alternative diagnosis in patientswith equivocal signs and symptoms. Use of this rapid, non-operator dependentand highly accurate examination may decreasedelays in appropriate medical or surgical therapy as well as unnecessary delayed observation.
